# ASTHMA IN THE OBESE: YET ANOTHER REASON TO LOSE WEIGHT

**DOI:** 10.4103/0970-2113.44128

**Published:** 2008

**Authors:** PR Gupta

**Affiliations:** Department of Chest & TB, Member Secretary, Medical Education Unit, SMS edical College, Jaipur

Over 1 billion people around the world are overweight.^1^ Indeed, obesity has now emerged as an important cause of morbidity and mortality in mankind. It is a significant risk factor for type 2 diabetes mellitus, hypertension, atherosclerosis, rheumatoid arthritis and cancer, more so when the weight gain is in and around the abdomen.^2^ Of late, it has been realised that obesity is a risk factor for asthma too. Further, it may alter the severity and control of asthma. Since the prevalence of both, obesity and asthma, is rising, it is now pertinent that all the respiratory physicians should have knowledge about this adverse relationship.

The effect of weight gain on asthma has been examined in several epidemiological studies in adults^3^ and children.^4^ Majority of these studies have shown an increased risk of incident asthma with an increase in body mass index (BMI). Importantly, obesity antedates asthma. Experimental data from studies in obese mice also support the causal relationship between obesity and asthma.

The major criticism of the epidemiological studies has been the basis of diagnosis of asthma. Obesity impairs ventilatory functions i.e. forced expiratory volume in one second (FEV_ 1_), forced vital capacity (FVC), total lung capacity and expiratory reserve volume.^5^ Besides, it may reduce respiratory muscle strength, decrease thoracic cage compliance and impede diaphragmatic excursion, especially when it is massive and central. The resultant increase in work of breathing may lead to the perception of increased respiratory effort i.e. dyspnoea. The latter may be confused as asthma. Thus, there is a risk of over diagnosing asthma in the obese. Further, it is apprehended that the correlation between obesity and asthma may not be causally related but may be due to existence of common risk factors.

Several mechanisms have been proposed to explain the biological basis for the relationship between obesity and asthma. Common genetics,^6^ obesity related increase in serum levels of pro-inflammatory adipokines^7^ or decrease in anti--inflammatory adiponectins^8^ and many of the obesity related bio-chemical changes/ co-morbidities may contribute.

Dixon et al^9^ noted the positive correlation between serum interleukin (IL-6) levels and asthma severity. Use of anti-IL-6 antibodies decreased airway responsiveness in obese but not in lean mice.^10^ Monocyte chemotactic proteins (MCPs), which polarise T cells from T helper type I (Th I) to Th2 phenotype, were found to be up regulated in the airways of obese asthma patients.^11^ Increase in levels of other adipokines like eotaxin, visfatin, complement, plasminogen activator inhibitor (PAI-I) and tumor necrosis factor α (TNFα) have also been implicated.^12^

Aerosol of ovalbumin caused marked increase in BAL eosinohils and Th2 cytokines in sensitized mice treated with buffer but not in those treated with full-length murine recombinant adiponectin,^13^ suggesting an anti-inflammatory role of the latter in asthma.

Increased serum leptin predicted incident asthma in boys^14^ and to some extent in women^15^ and exogenously administered leptin augmented ozone or allergen induced airway inflammation,^16^ suggesting its role. Hypercholesterolemia may also contribute, acting independently or in combination with other factors. Sleep disordered breathing (SDB) and gastro-esophageal reflux disease (GERD), which are induced by obesity, may cause asthma independent of the parent disorder. Relationship, if any, between asthma and other common obesity related conditions such as type 2 diabetes, atherosclerosis and hypertension is yet to be studied.

Female sex hormones have also been implicated^17^ as the effects of obesity on asthma are greater in women than men.

Conflicting data are available as to whether obesity increases asthma severity. An increase in severity of asthma with incident obesity was observed by Akerman et al^18^ but Lavoie et^19^ could not find such a correlation. Cassol et al^20^ found it only in women.

Perception of dyspnoea in an obese may be due to obesity related changes on the respiratory system or the related co-morbidities like GERD. Therefore, a symptom based diagnosis of asthma or its severity is not enough and an objective diagnosis of asthma based on spirometry is mandatory in an overweight patient.

Asthma is more difficult to control in obese than lean asthma patients. Thus, Saint-Pierre et al^21^ found that compared to lean patients, overweight patients who were initially poorly controlled, were more likely to remain so, despite guideline based treatment. Obesity related changes in drug distribution and duration of effect, interactions between some therapeutic agents used to treat co-morbidities like SDB and GERD and the altered hormonal environment of the obese patient may be responsible for this infirmity. Peters-Golden et al^22^ observed that the response to montelukast increased with BMI suggesting its role in treatment of obese asthma patients. Relative risk for an exacerbation was greater in obese asthma patients treated with low dose theophylline as compared to lean individuals suggesting a possible contraindication for the drug.^9^ Thus there is a clear need to improvise new therapeutic strategies for these patients, including the simultaneous control of comorbidities like GERD.

Novel therapeutic approaches are being considered in obese asthma patients. Thiazolidinediones (used in type-2 diabetes mellitus) increase serum adiponectin levels. It reduceds airway inflammation in animal models of asthma^23^ and is worth evaluation. Etanercept (soluble TNFα receptor)^24^ and infliximab (TNFα antibody)^25^ have been shown to improve airway function or reduce exacerbations in asthma patients. Anti immunoglobuline (IgE) may also find a place in management of asthma in obese.

Surgically induced weight reduction has resulted in significant improvements in asthma prevalence, severity, medication use, and hospitalizations in adults.^26^ In another study, diet induced weight loss also augmented airway function in obese asthma patients.^27^ Therefore, the best therapeutic strategy for obese asthma patient is to lose weight. The effect of weight control on asthma in children remains to be established.

Is asthma in obese preventable? Data on development of asthma in children indicate that obesity is a risk factor for asthma during early childhood.^28^ It is, therefore, conceivable that interventions aimed at controlling weight gain during first few years of life may be effective in reducing or at least delaying the incident asthma. Studies are urgently needed to unravel this issue of vital importance. Till then, the BMI of the patient should form an important consideration while prescribing asthma medications.
